# Crystallization Kinetics and Structural Properties of the 45S5 Bioactive Glass and Glass-Ceramic Fiber Doped with Eu^3+^

**DOI:** 10.3390/ma13061281

**Published:** 2020-03-12

**Authors:** Agata Baranowska, Magdalena Leśniak, Marcin Kochanowicz, Jacek Żmojda, Piotr Miluski, Dominik Dorosz

**Affiliations:** 1 Department of Power Engineering, Photonics and Lighting Technology, Bialystok University of Technology 45D Wiejska Street, 15-351 Bialystok, Poland; a.baranowska@pb.edu.pl (A.B.); j.zmojda@pb.edu.pl (J.Ż.); p.miluski@pb.edu.pl (P.M.); 2 Faculty of Materials Science and Ceramics, AGH University of Science and Technology, 30 Mickiewicza, Poland; mlesniak@agh.edu.pl (M.L.); ddorosz@agh.edu.pl (D.D.)

**Keywords:** thermal analysis, glass-ceramics, bioactive glass fibers, luminescence

## Abstract

An investigation of the crystallization kinetics of 45S5 Bioglass^®^ using differential scanning calorimetry is presented in this paper. Thermal analysis was performed using the Friedman method. The activation energy and the Avrami index were calculated. The glass samples were subjected to additional controlled heat treatment at 620 °C in order to obtain bioactive glass-ceramics with enhanced mechanical properties. X-ray powder diffraction (XRD) measurements indicated the formation of the glass-ceramic structures of three cyclosilicates: Na_4_Ca_4_(Si_6_O_18_) or Na_6_Ca_3_(Si_6_O_18_) or Na_16_Ca_4_(Si_12_O_36_). Based on middle infrared region (MIR) results, it can be concluded that the crystalline phase present in the tested materials was Na_6_Ca_3_(Si_6_O_18_) (combeite). Material was doped with Eu^3+^ ions, which act as a spectroscopic probe for monitoring the structural changes in the glass matrix. The decreasing value of the fluorescence intensity radio parameter indicated symmetry around the europium ions and, thus, the arrangement of the glass structure. The bioactive properties of the examined glass-ceramics were also determined. The bioactive glass fibers doped with Eu^3+^ were manufactured using two different methods. Its structural and luminescent properties were examined.

## 1. Introduction

Since 1969, 45S5 Bioglass^®^ has been the first and the best know bioactive glass and it is widely used as a bone and tissue replacement because of its excellent biocompatibility [1,2,3]. It is classified as a class A bioactive glass, which means that this material is osteoconductive and osteoproductive and exhibits the highest rate of hydroxyapatite (HA) formation in vitro and in vivo that are necessary for tissue engineering applications [4,5,6]. However, its main disadvantages are poor mechanical strength and low fracture toughness, which limits use to low-load applications [7]. Controlled heat treatment allows the formation of crystalline phases within the glass structure, which could enhance its mechanical quality [7,8,9,10]. On the other hand, the crystallization process can change the bioactive properties but rarely leads to creating completely inert material [11,12]. Glass-ceramics are characterized by lower surface reactivity than typical glasses because of the decreasing of the Si-OH groups on their surfaces. Calcium ions are trapped in crystalline phases, which limit their release [13]. Besides the typical and most commonly used methods of measuring the structure of glass and glass-ceramic materials, there is one called a “spectroscopic probe”, which allows quick verification of the glass structure. The material local environment can be determined by doping the glass with Eu^3+^ ions and analyzing the changes of photoluminescence intensity [14]. The degree of asymmetry around europium ions can be determined by the interpretation of the hypersensitive transition ^5^D_0_→^7^F_2_ (the ratio between two luminescence bands at 613 nm (^5^D_0_→^7^F_2_) and 591 nm (^5^D_0_→^7^F_1_)). Another aspect of the doping of bioactive glasses and fibers is the possibility to measure their degradation by monitoring in time the intensity of the luminescence signal [15]. An example of the use of optical measurements is the research of J. Massera et al. [16,17]. They described the changes in fiber light transmission as a consequence of changes in the fiber structure and created Ca-P layer. It is worth pointing out that the only a few attempts of obtaining glass fibers from the 45S5 glass were shown [18,19,20]. Moreover, that fibers were obtained by the “hand-drawn method from the melt”, which influences their mechanical and geometrical properties. Additionally, the high tendency of crystallization of 45S5 glass significantly impedes a fiber drawing process. The proposed research is directed to move a step toward obtaining 45S5 fibers by the traditional drawing method and alternative method (drawing from the glass melt). The main advantage of the glass fibers manufactured by the drawing method is that their diameter can be precisely controlled and the fibers with a length of tens of meters can be obtained with ease. Bioactive glass fibers can be used as an excellent reinforcing element in composite structure [21,22].

In this work the thermal, mechanical, and structural properties of the 45S5 Bioglass are presented. As a result of the controlled heat treatment of the glass, a glass-ceramic material was obtained. Based on Differential Scanning Calorimetry (DSC) measurements, with a heating rate of 5, 10, and 15 °C/min, the kinetics of the crystallization was determined. Activation energy and the Avrami index were calculated. XRD measurements showed the crystalline phases in the glass-ceramics. Deconvolution of the middle infrared region (MIR) spectra and the assignment of component bands to the corresponding vibration allowed us to establish that the glassy phase of the analyzed materials was a silicate‒phosphate network. The presence of Na^+^ and Ca^2+^ cations in the structure of the analyzed materials (glass and glass-ceramic) contributed to the depolymerization of the silicate‒phosphate network. The mechanical properties of the glass were improved, and thus the biological properties were not interfered with. It is important to mention that the high tendency of 45S5 glass to crystallize makes a fiber drawing process not easy, which will be the following step of the author’s investigations. A detailed analysis of the crystallization kinetics will allow for the optimization of the fabrication of a 45S5 fiber.

## 2. Materials and Methods

The glass was made of high purity materials (Sigma Aldrich) by a standard method consistent with the following molar composition: 46.1SiO_2_−24.4Na_2_O−26.9CaO−2.6P_2_O_5_. The homogenized set was melted at T = 1400 °C for 30 min in a platinum crucible placed in an electric furnace (CZYLOK Company, Jastrzębie-Zdrój, Poland) under an oxide atmosphere. To obtain the required rod shape, the molten glass was poured into a stainless-steel mold with a diameter of 10 mm. Then the material was annealed at 520 °C for 12 h to reduce thermal stress. The transparent and homogeneous glass rod was cut into 2 mm disc plates. The thermal properties of the fabricated glass were determined using Differential Scanning Calorimetry (DSC) from SETARAM Labsys (Setaram Instrumentation, Caluire, France) at a temperature range of 40–1100 °C in a nitrogen atmosphere. DSC measurements were collected at several heating rates: 5, 10, and 15 °C/min. Crystallization kinetics were designated using AKTS Thermokinetics Software (version 2.56, AKTS AG, Sierre, Switzerland) and calculated with the isoconversional Friedman method. Furthermore, the Avrami index was determined. Fabricated glass samples were subjected to additional heat treatment in an electric tube furnace (CZYLOK Company, Jastrzębie-Zdrój, Poland) in an ambient air atmosphere for 1, 2, 4, 8, and 16 h at 620 °C. After a given heating period, the samples were allowed to cool rapidly in static air. The microhardness was measured using the Vickers method. The formed crystallites were examined by the X-ray powder diffraction (XRD) method in the range of 10° to 80° using an X’Pert Pro diffractometer (PANalytical, Eindhoven, Netherlands). The Cu X-ray tube with K_α_ radiation was used. The formed nanocrystals of the obtained glass-ceramics calculated from Scherrer’s [23] formula (Equation (1)) had a size of approximately 29 nm.

(1) Dp=0.94λβ12cosθ

where *D_p_* is the average crystallite size, *β* is the line broadening in radians, *θ* is the Bragg angle and *λ* is the X-ray wavelength.

Fourier-Transform Infrared Spectroscopy (FTIR) were recorded with a Bruker Company Vertex 70v spectrometer (Rheinstetten, Germany). The samples were prepared using the pellet method in KBr. The absorbance spectra were analyzed after 128 scans at the resolution of 4 cm^−1^ in the range of 1400–400 cm^−1^ (MIR—middle infrared region). MIR spectra were normalized and then deconvoluted using Fityk software (0.9.8 software, Marcin Wojdyr, Warsaw, Poland, open-source (GPL2+)). The coefficient of determination (R square) of all the deconvoluted FTIR spectra was 0.99. The standard deviation of the position and full width at half maximum (FWHM) of each of the component bands was 4 cm^−1^. The bioactivity test was executed using a known method, i.e., by immersing the samples with dimensions of 5 mm × 5 mm × 7 mm (the mass of the glass was 0.473 g) for 7 days in 30 mL of Simulated Body Fluid (SBF) at a temperature of 37 °C, prepared as by Kokubo [24,25] in static conditions. The glass and glass-ceramic morphology was observed by a FEI Company Nova Nano SEM 200 scanning electron microscope (Hillsboro, OR, USA) and the analyses were performed in the secondary electron mode (SE). Before these measurements, the samples were covered with a 5 nm carbon layer. The carbon layer was deposited using the Radio Frequency Sputtering technique using the Unitra Unima sputter (Unitra Unima, Warsaw, Poland). Next, the 45S5 glass was doped with 0.2 mol% Eu_2_O_3_ at the expense of the silicon content (45.9SiO_2_−24.4Na_2_O−26.9CaO−2.6P_2_O_5_–0.2Eu_2_O_3_ (%mol)). The luminescence spectra were collected by a Stellarnet Green-Wave monochromator (Stellarnet Inc., Tampa, FL, USA) in the range of 500–700 nm with 0.5 nm resolution and under 396 nm laser diode excitation. The luminescence signal was collected by transmitting an optical fiber with numerical aperture: NA = 0.5 and 400 µm in diameter. Bioactive glass fibers were fabricated by two methods: the standard method, i.e., drawing method (rod-in-tube method) and an alternative method—drawing from the glass melt. The second method was proposed by Crupper et al. [18]. The high content of calcium and sodium increases the susceptibility of the structure to crystallization, which significantly limits the possibility of fiber production and worsens the mechanical properties. This method involves drawing fibers from the glass melt as soon as the crucible is removed from the furnace. In the standard method of drawing fibers, the molted glass is poured into a stainless-steel form to obtain a glass rod 10 mm in diameter, which is then annealed in an air atmosphere, as was written before. The glass fibers are manufactured by means of SG control 7.5 m in a height drawing tower. A glass rod is fed into the tube furnace (SG Control, Newton, UK) with a sufficiently narrow temperature zone at a speed of 0.2–1 mm/min. Then the glass is softened and flows out through the furnace outlet. The outgoing stream of glass is drawn into a fiber using a rotating drum. The required diameter is adjusted by the speed of the drum and the feeding speed of the fiber preform. The drawing temperature depends on the glass composition, in this case, it was 900–1000 °C. 

## 3. Results and Discussion 

### 3.1. Thermal Analysis

**Figure 1:**
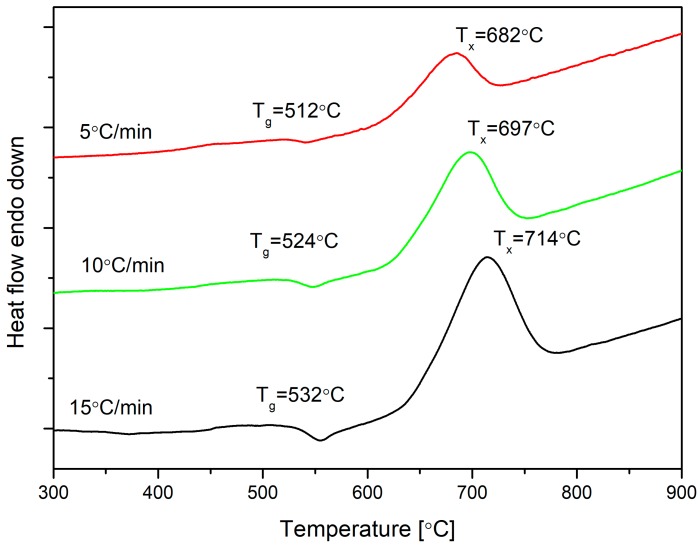
Differential Scanning Calorimetry (DSC) curves recorded for 45S5 Bioglass^®^ at heating rates of β = 5, 10, and 15 °C/min.

Crystallization kinetics research of the Bioglass^®^ was obtained as a function of heating rates: 5, 10, and 15 °C/min. DSC results of the melted 45S5 are shown in Figure 1. As a result of increasing the heating rate, the concentration of nucleation sites decreased due to a shorter period of time in the nucleation range and, consequently, the crystallization peak occurred at a higher temperature, which is associated with the lower melt viscosity.

The glass transition temperature (T_g_) depended on the structural parameters and its value increased with a higher heating rate; the same situation is with crystallization temperature, T_x_. In Table 1, ∆T= (T_x_ − T_g_) is presented, and it is named as a thermal stability parameter. In optical fiber technology, parameter ΔT above 100 °C describes glass as a suitable material for glass fiber drawing [26]. Here, the parameter was included in the range from 161 to 182 °C, but from the experience of authors and literature reports [27,28,29], it is difficult to draw the fiber. The phenomenon of a rapid crystallization process can be observed in the recorded DSC curves (Figure 1). It was related to a high concentration of sodium and calcium in this glass. 

**Figure 2:**
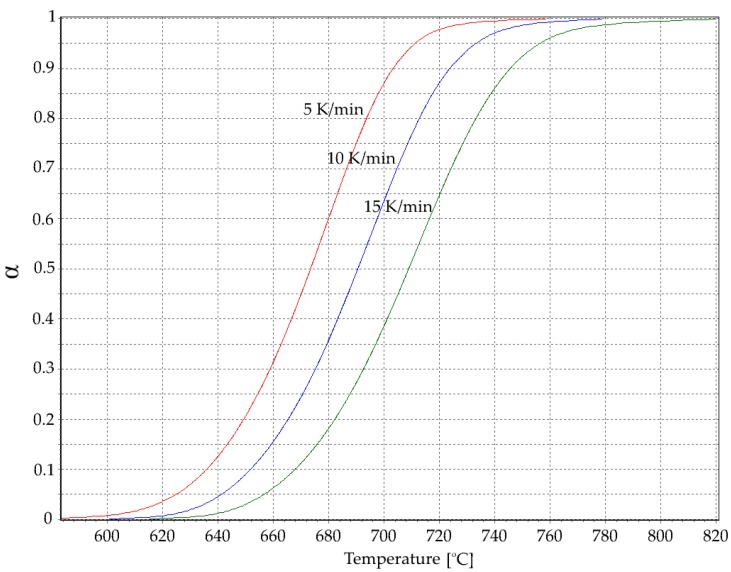
Degree of transformation α as a function of temperature at different heating rates.

α–T dependence at different heating rates (Figure 2) was calculated according to the equation

(2) $$\alpha \left(t\right)=\frac{\int_{t_{o}}^{t}\left(S\left(t\right)-B\left(t\right)\right)dt}{\int_{t_{o}}^{t_{end}}\left(S\left(t\right)-B\left(t\right)\right)dt}$$

where t_0_ is the start of the analysis, t_end_ is the end of the analysis, B(t) is the baseline, and S(t) is the signal DSC.

**Figure 3:**
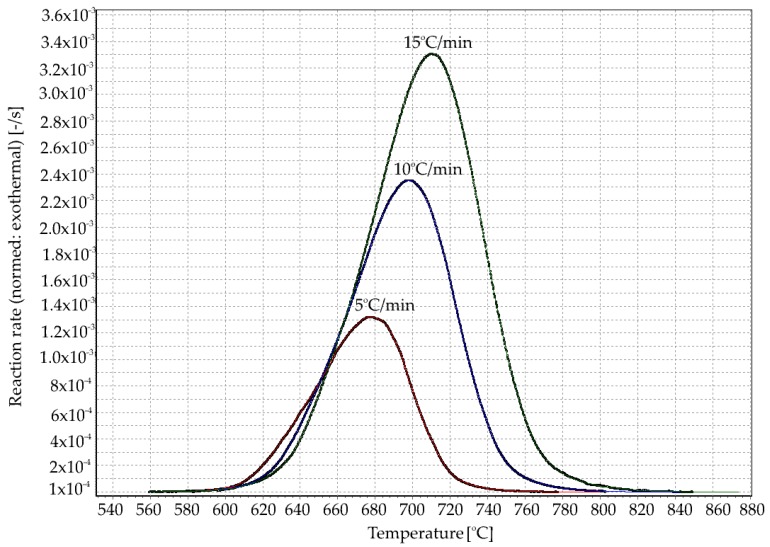
Conversion rates as a function of the temperature at heating rates β_i_= 5, 10, and 15 °C/min.

The reaction rate as a function of the temperature at different heating rates was calculated using formula (3) and shown in Figure 3.

(3) $$\frac{d\alpha }{dt}=\frac{\left(S\left(t\right)-B\left(t\right)\right)}{\int_{t_{o}}^{tend}\left(S\left(t\right)-B\left(t\right)\right)dt}$$

#### Friedman Analysis

The linear differential isoconversional method suggested by Friedman [30] is based on the Arrhenius equation

(4) $$\frac{d\alpha }{dt}=A\;exp\left(-\frac{E}{RT}\right)f\left(\alpha \right)$$

where *t* (s) it the time, *T* (°C) is the temperature, *α* is the degree of conversion that represents the volume of the crystallized fraction, *E* (kJ mol^−1^) is the activation energy, *A* (s^−1^) is the pre-exponential (frequency) factor, *R* (J mol^−1^ K^−1^) is the universal gas constant, and *f(α)* is the differential conversion model (reaction model).

Under non-isothermal conditions with a constant heating rate of β = dT/dt, Equation (4) can be transformed as

(5) $$\frac{d\alpha }{dT}=\frac{d\alpha }{dt}\left(\frac{1}{\beta }\right)=\frac{A}{\beta }\exp\left(-\frac{E}{RT}\right)f\left(\alpha \right)$$

**Figure 4:**
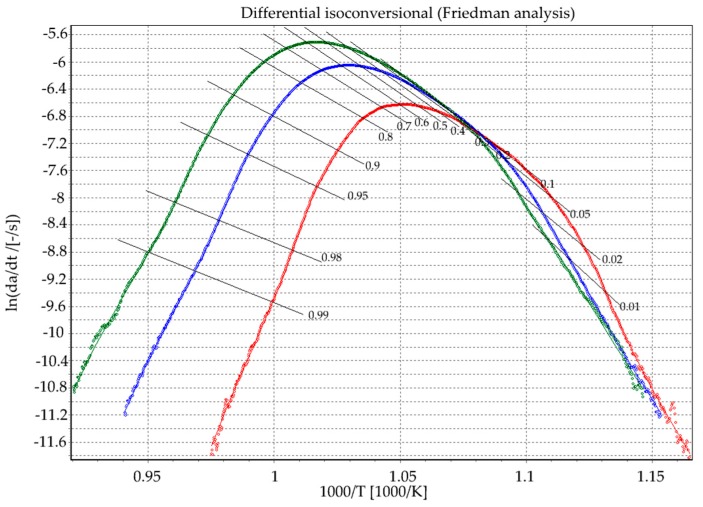
Isoconversional plots of ln(dα/dt) versus 1000/T according to the Friedman method (with β_i_ = 5, 10, and 15 K/min).

For several heating rates, *β_i_*, the Friedman method can be obtained as follows directly from Equation (4), at specific crystallization fraction *α*:

(6) $$ln{\left(\frac{d\alpha }{dt}\right)}_{\alpha i}=C_{F}\left(\alpha \right)-\frac{E_{\alpha }}{RT_{\alpha i}}$$

where the subscript *i* indicates different heating rates and the *C_F_(α) = ln(A_α_f(α))*. For a crystallization fraction α value and several heating rates, β, the pairs of (dα/dt)_αi_ and T_αi_ were determined experimentally from the DSC diagram (Figure 1). The parameters *E_α_* and *C_F_(α)* at this definite value of *α*, were estimated from a plot of ln(dα/dt) _αi_ versus 1/T_αi_ across at least three different heating rates (Figure 4). This method can be used to calculate the local activation energy of crystallization E_A_, at specific α by means of different heating rates. 

**Figure 5:**
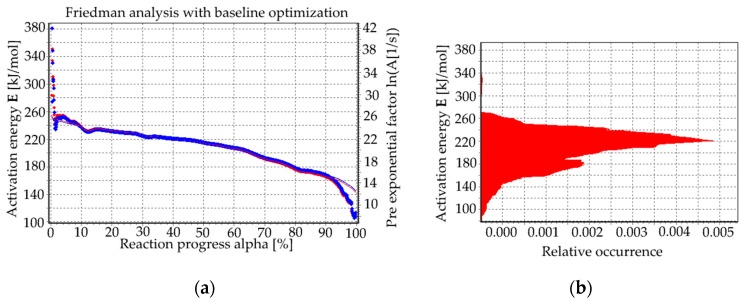
(**a)** Activation energy as a function of the reaction progress; (**b**) histogram of the activation energy.

The logarithm of the reaction rate over 1000/T resulted in a straight line with the scope E/R in a full range of conversion degree (Figure 4). The local activation energy could be determined by the slopes of the lines that were drawn through isoconversional points at different heating rates for certain reaction progress. This parameter determined the tendency of the crystallization of the glass. In fabricated glasses, the average activation energy amount was 220 kJ/mol (Figure 5), which is similar to those calculated by J. Massera et al. 230 ± 30 kJ/mol [29] or L. Lefebvre et al. [8]. Figure 5a presents the activation energy value and pre-exponential factor as a function of the reaction progress. In Figure 5b the histogram of the activation energy is presented, i.e., the activation energy as a function of the relative occurrence (approximately, for 230 ± 30 kJ/mol the occurrence value was the highest).

The Avrami number was used for deducing the mechanism of phase transformations that occur via nucleation and the crystal growth mechanism and can be calculated from Augis and Bennett’s [31,32] formula,

(7) $$n=\left(\frac{2.5}{FWHM}\right)\frac{RT_{x}^{2}}{E}$$

where *FWHM* is the full width at half crystallization peak maximum. Thus, the Avrami exponent *n* for our system at T_x_ = 682, 697, and 714 °C were 1.53, 1.42, and 1.34, respectively (Table 2). The average Avrami parameter for the three measurements was therefore 1.43 ± 0.09. Such a value indicates a surface and two-dimensional crystallization in the examined glass [33]. 

### 3.2. Structure Study 

**Figure 6:**
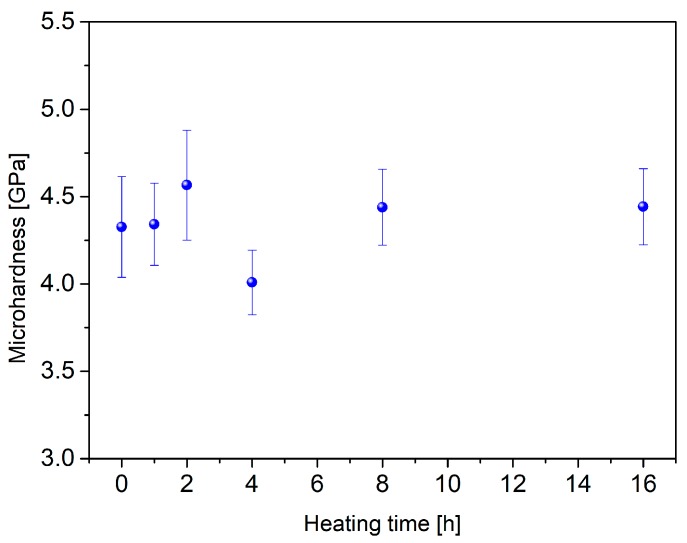
Microhardness values of the heat-treated glass samples.

In order to obtain glass-ceramics, the glass samples were subjected to a process of additional heat treatment at 620 °C at specific periods of time (1, 2, 4, 8, and 16 h). Afterward, the microhardness was measured. The highest microhardness (4.6%) was obtained (Figure 6) and the value was similar to this in literature 4.7 ± 0.5 GPa [34]. 

**Figure 7:**
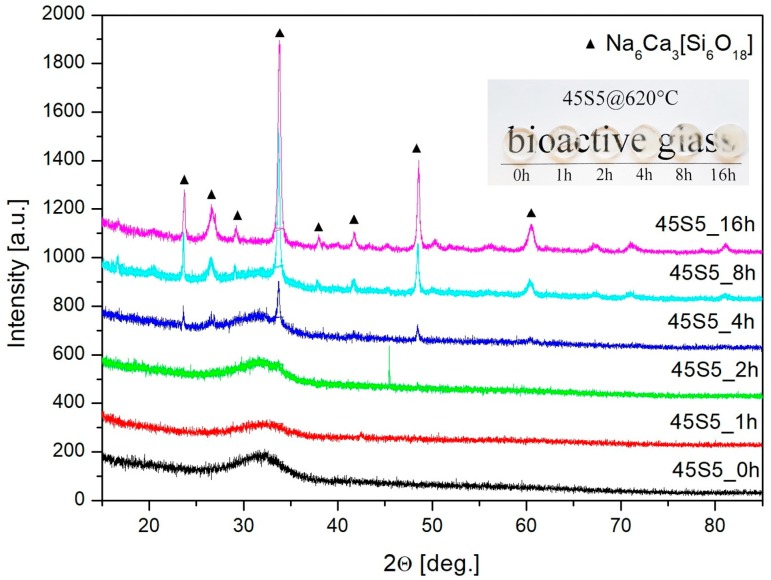
XRD patterns and samples photo (inset) of fabricated 45S5 glass as melted and annealed at 620 °C for 1, 2, 4, 8, and 16 h.

The diffraction patterns of samples were presented in Figure 7. The XRD measurements confirmed the presence of the crystalline phase and only after 4 h of heating the 45S5 glass there were clear reflections present on the amorphous halo. With an extension of the heating time, the proportion of the crystalline phase increased at the expense of the amorphous phase—the disappearance of the amorphous halo. Unfortunately, the phase analysis was not entirely clear, because it indicated the possible presence of up to three cyclosilicates: Na_4_Ca_4_(Si_6_O_18_) (JCPDS 04-008-0810) and/or Na_6_Ca_3_(Si_6_O_18_) (JCPDS 04-012-8759) and/or Na_16_Ca_4_(Si_12_O_36_) (JCPDS 04-015-4977). The first two were cyclosilicates in structures of which there were isolated 6-membered silico‒oxygen rings with different symmetry and, in the last, isolated 12-membered rings. Their distinction based on XRD research is complicated, especially in the case of dealing with glass-crystalline material. MIR spectroscopy is useful for resolving this problem, because characteristic bands are present in the MIR spectra of cyclosilicates, whose position and intensity depend on the number of members and the symmetry of the silico‒oxygen rings [35,36,37].

Knowledge of the structure of the glass and the crystalline phases of the obtained materials is very important as both the glassy matrix and the amount of type of crystalline phases determine the final usage parameters of the glasses and glass-ceramic materials. Determining the type of the crystalline phase is usually quite simple using the X-ray powder diffraction (XRD) technique, however, (as shown above in the X-ray phase analysis part) it also creates many problems, especially in the case of glass-crystalline materials. In the case of glassy and glass-ceramic materials, due to the presence of an amorphous phase, which is characterized by the lack of long-range order, it is necessary to use methods for determining the so-called near and middle-range ordering. From this point of view, spectroscopic methods are the most suitable, in particular mid-infrared spectroscopy (MIR).

According to the literature, the structure of silicate‒phosphate glasses consists of connected (SiO_4_)^4−^ and (PO_4_)^3−^ tetrahedrons, forming a three-dimensional network, in which each silicon ion is bound to four other silicon or phosphorus ions by bridging oxygen ions (Si-O-Si, Si-O-P—bridging bonds), while each phosphorus ion has only three bridging bonds [38,39]. In the fourth corner of the (PO_4_) tetrahedron, there is a non-bridging oxygen ion bound to the central phosphorus ion by a double bond (O=P). This oxygen ion cannot be a bridge for another tetrahedron, therefore, it does not participate in the creation of polymerized anions. However, it can create bonds with modifier cations of the network, which results in the formation of M-O-PO_3_ bonds, (M—cations such as K^+^, Ca^2+^, Mg^2+^, etc.). The introduction of modifiers of the network to the structure of silicate—phosphate glasses also leads to breaking the Si-O-Si, Si-O-P, and P-O-P bonds. As a result, there is depolymerization of the silico—phosphate network—the appearance of broken bridges—Si-O^−^ and P-O^−^ (terminal bonds) [35,40,41,42,43].

In the case of silicate‒phosphate glasses the use of the silico‒oxygen (SiO_4_)^4−^ and phospho‒oxygen (PO_4_)^3−^ tetrahedron model as monomeric units recreating the network, allows for quite precise interpretation of the spectra in the mid-infrared range (MIR). Therefore, according to the literature, in the MIR spectra of silicate‒phosphate glasses in the 1400–400 cm^−1^ range appear bands that are listed in Table 3. 

**Figure 8:**
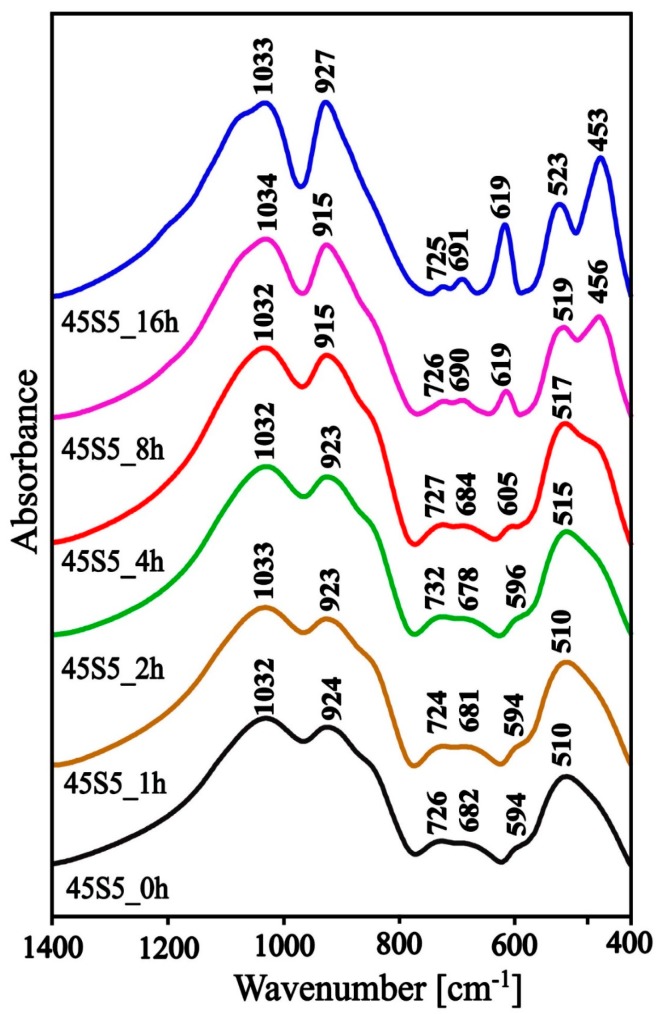
Middle infrared region (MIR) spectra of the 45S5_0h glass and glass-ceramic samples heated in 620 °C at times of 1, 2, 4, 8, and 16 h.

In order to determine the structure of the initial silicate‒phosphate glass (45S5_0h) and the effect of different heating times (1, 2, 4, 8, and 16 h at 620 °C—temperature determined on the basis of the DSC) on its structure and phase composition, mid-infrared tests were performed for all samples. The MIR spectra in the range of 1400–400 cm^−1^ are shown in Figure 8. All spectra in Figure 8 were similar—there were bands characteristic of silicate‒phosphate glasses in all spectra (Table 3). In the spectra of glass-ceramics labeled as 45S5_8h and 45S5_16h, a distinct band appeared at about 620 cm^−1^ (Figure 8). The presence of such an intense band at approximately 615 cm^−1^ unambiguously indicated the presence of 6-membered silico‒oxygen rings with high symmetry. On this basis, it can be concluded that the crystalline phase present in the tested materials is Na_6_Ca_3_(Si_6_O_18_) (combeite). This band was not observed in the spectrum of the 45S5_4h sample, although the presence of a cyclosilicate was found on the basis of XRD studies. Most likely, it was associated with a small content of the Na_6_Ca_3_(Si_6_O_18_) in the sample, below the detection limit of the MIR. In Figure 8 it can be observed that the band at approximately 510 cm^−1^ (45S5_0h, 45S5_1h, 45S5_2h, 45S5_4h) was split into two bands, approximately at about 520 and 460 cm^−1^ (45S5_8h, 45S5_16h). These bands were assigned to the bending vibrations of ^-^O-Si-O^-^ and (Si)O-Si-O(Si), respectively [1,2,3,4]. In the analyzed glass-ceramic materials (45S5_8h and 45S5_16h) these were characteristic bands for Na_6_Ca_3_(Si_6_O_18_) cyclosilicate [37].

**Figure 9:**
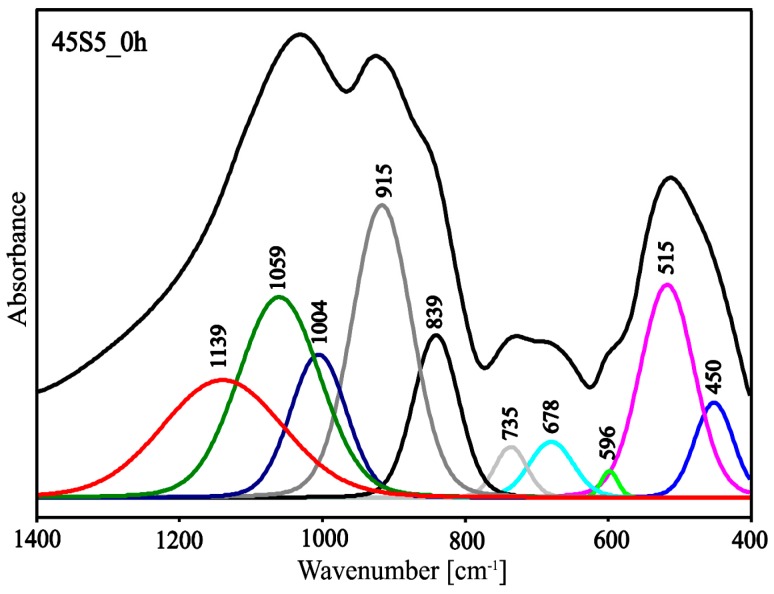
Deconvoluted MIR spectrum of the 45S5_0h sample.

**Figure 10:**
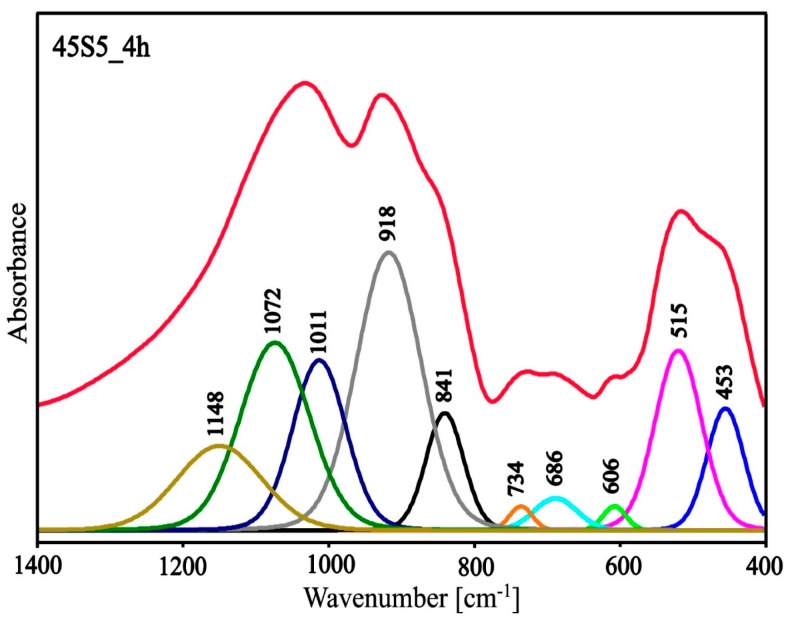
Deconvoluted MIR spectrum of the 45S5_4h sample.

Due to the large half-width of the bands in the spectra (Figure 8), it was difficult to precisely assign all bands to the appropriate types of vibrations. To facilitate interpretation, the deconvolution of the MIR spectra of the 45S5_0h and 45S5_4h samples into component bands was performed. The resulting deconvolution is presented in Figure 9 and Figure 10. The parameters of the component bands together with the assignment to the corresponding vibrations are shown in Table 2 and Table 3.

Based on the deconvolution of the MIR spectra and then the component bands being assigned to the corresponding vibration it can be established that the glassy phase of the analyzed materials was a mixed silicate‒phosphate network (Table 3, Table 4 and Table 5). The presence of the Na^+^ and Ca^2+^ cations in the structure of the analyzed materials resulted in the depolymerization of the silicate‒phosphate network (Figure 2 and Figure 3). As glass was heated, a rapid shift in the position of the band from 1059 (45S5_0h) to 1071 cm^−1^ (45S5_4h) was seen. This was accompanied by a significant increase in the integral intensity of the band characteristic for 6-membered silico‒oxygen rings (at approx. 600 cm^−1^). This was related to the appearance of the first crystals of the combeite (Na_6_Ca_3_(Si_6_O_18_)) (XRD results). As already mentioned above, the integral intensity of the band characteristic for isolated 6-membered rings (at approx. 615 cm^−1^) presented in the structure of the Na_6_Ca_3_(Si_6_O_18_) increased rapidly when the heating time was increased to 16h (Figure 8)—increasing the amount of the Na_6_Ca_3_(Si_6_O_18_). This confirmed the constant increase in the ordering of the system as a result of the thermal treatment. 

### 3.3. Bioactivity Test

**Figure 11:**
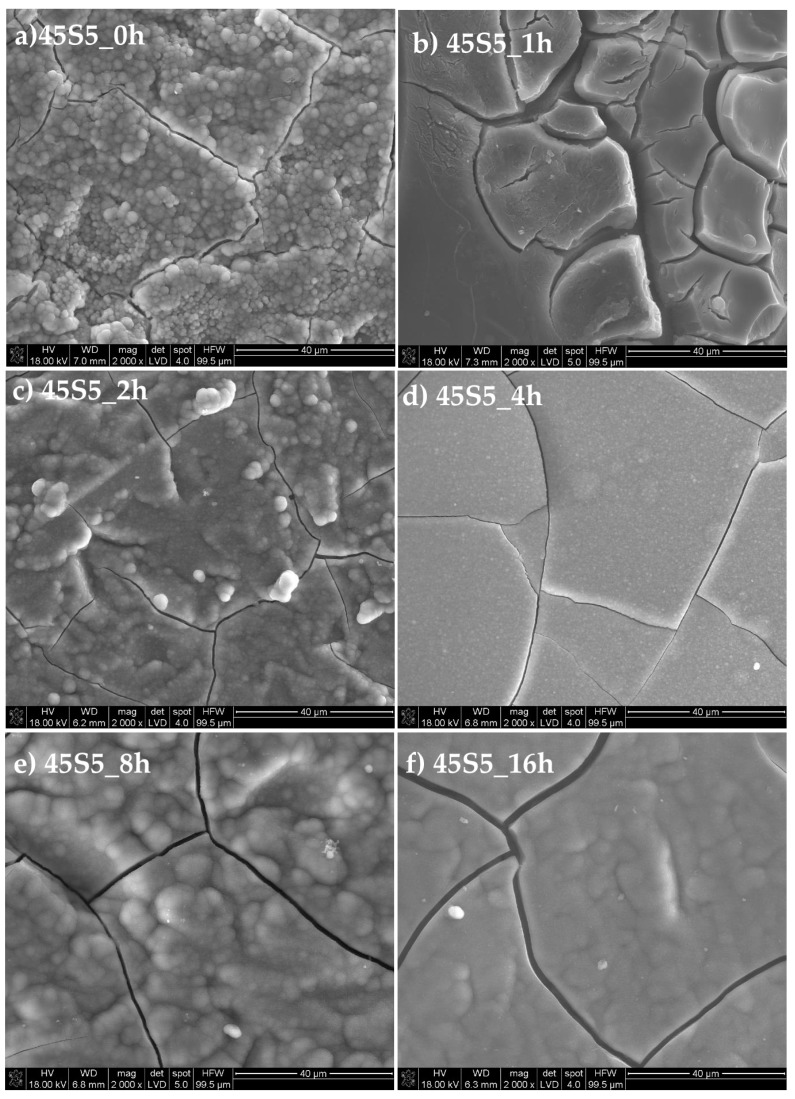
SEM micrographs of 45S5 annealed at 620 °C for (**a**) 0 h, (**b**) 1 h, (**c**) 2 h, (**d**) 4 h, (**e**) 8 h, and (**f**) 16 h. Accelerating voltage—18 kV.

An in vitro bioactivity test was carried out as proposed by Kokubo in 1991 [24]. The heat-treated (for 1, 2, 4, 8, and 16 h in 620 °C) 45S5 samples, after immersion in Simulated Body Fluid (SBF) for 7 days, are presented in Figure 11. Incubation in solution changed their surface morphologies. The following reactions at the surface: ion exchange with SBF, hydration, hydrolysis, and condensation caused the formation of a silica-rich layer, which can be porous with water and silanol groups. When the dissolution of the ions exceeded then the amorphous calcium phosphate film crystallized into a hydroxy-carbonate apatite (HCA) layer [44]. The HCA formed on the surfaces of the examined glasses after immersion. The pictures (Figure 11) of the microstructure show that the partial crystallization of the glasses does not define the loss of their bioactivity, and a typical HCA shape structure was observed [45].

### 3.4. Luminescent Properties of Glass and Glass-Ceramics

The glasses doped with lanthanide ions were characterized by their luminescence spectra in the UV-VIS (Ultra Violet – Visible) range. However, doping the glasses allowed the determination of the surrounding structure of rare-earth ions by measuring the specific emission spectra [46]. The 45S5 Bioglass^®^ was doped with 0.2 (%mol) europium ions and then, after heat treatment at 620 °C for 1, 2, 4, 8, and 16 h, the luminescence spectra were measured. The glass was excited by the 396 nm laser diode. 

**Figure 12:**
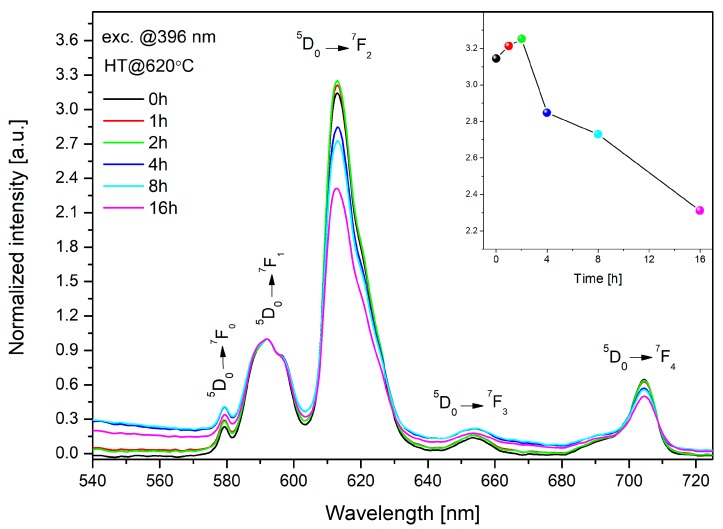
The characteristic of luminescence changes for 45S5 glass doped with Eu^3+^ ions before and after heat treatment at 620 °C. Asymmetry ratio of 45S5 bioactive glass doped with Eu^3+^ (inset).

Figure 12 presents the influence of the heat treatment of the doped bioactive glass on its luminescence profile and, thus, its structural properties. Eu^3+^ ions were used as a spectroscopic probe. Due to the host independent feature of ^5^D_0_→^7^F_1_ transition, the luminescence spectra were normalized at a wavelength of 591 nm. All samples were characterized by the highest emission at the wavelength of 611 nm originated from ^5^D_0_→^7^F_2_ “hypersensitive” transition. 

The ratio between the electric dipole transition (^5^D_0_→^7^F_2_) and magnetic dipole transition (^5^D_0_→^7^F_1_) determines the local inversion symmetry in the vicinity of europium ions, as follows [14]:

(8) $$R/O=\;\frac{I\left({}^{5}D_{0}\to {}^{7}F_{2}\right)}{I\left({}^{5}D_{0}\to {}^{7}F_{1}\right)}=\frac{I_{613\;\mathrm{nm}}}{I_{591\;\mathrm{nm}}}$$

The values of the asymmetry ratio were shown in Figure 12 (inset). It is worth noticing that above 4 h of annealing time, this ratio decreased significantly from 3.25 to 2.3. An increase of the symmetry around europium ions and observed crystalline phases form XRD patterns (Figure 7) indicated a less covalent bond between Eu^3+^ ions, which confirmed the creation of glass-ceramic material. 

A thorough analysis of the crystallization kinetics allowed a fiber to be drawn from the 45S5 bioactive glass. Based on the bulk glass measurements, the 45S5 Bioglass^®^ was doped with Eu^3+^. Manufacturing of the fibers was carried out by two different methods—(1) the standard method, i.e., drawing method (rod-in-tube method); (2) an alternative method, i.e., drawing from the glass melt.

**Figure 13:**
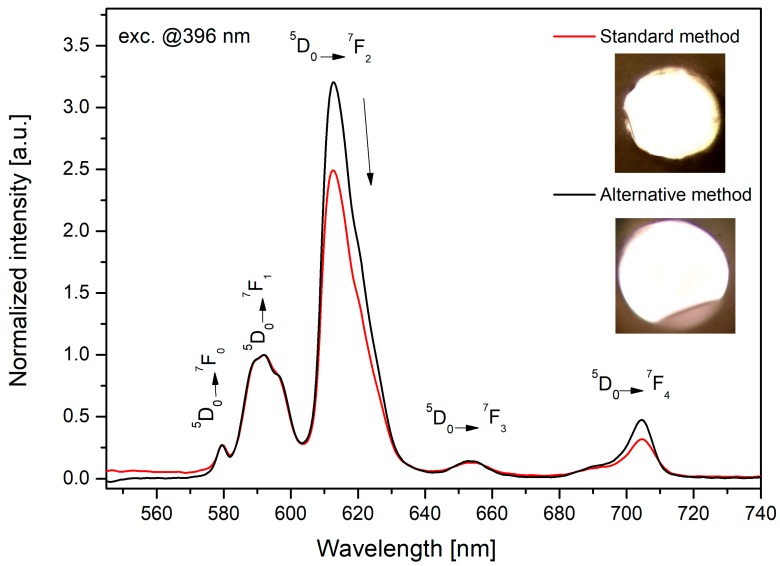
The characteristic of luminescence changes and photos (inset) for 45S5 fibers doped with Eu^3+^ drawn by a standard and alternative method.

**Figure 14:**
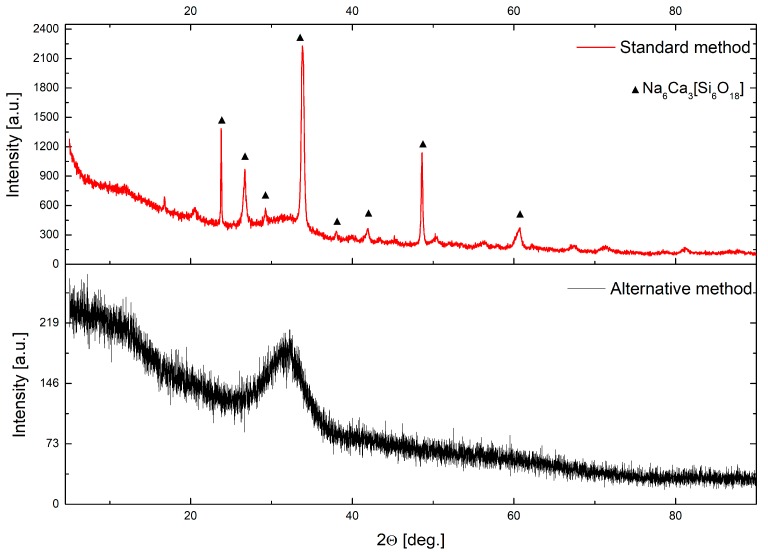
XRD patterns of fabricated 45S5 glass fiber drawn by a standard and alternative method.

Figure 13 presents the luminescence changes for the 45S5 fibers doped with Eu^3+^ manufactured by the two methods. The insets of Figure 13 represent the foreheads of the fibers with a 50µm diameter. The fiber obtained by the standard method was partially crystallized, which can be seen on its rough side surface presented in the photo, in contrast to those produced by the alternative method. The ratio between transition: ^5^D_0_→^7^F_2_ and ^5^D_0_→^7^F_1_ shows the local inversion symmetry in the vicinity of europium ions. An increase of the symmetry was observed in fiber produced by the standard method, which correlated with the partial crystallization of the structure and was proven by the XRD pattern (Figure 14). This study confirmed that the same crystalline structure (Na_6_Ca_3_(Si_6_O_18_)) as in glass was formed in the prepared fibers, drawn by the standard method. 

## 4. Conclusions

The kinetics of the crystallization of the fabricated 45S5 glass were analyzed by the Friedman method and the Avrami index was calculated as 1.43 ± 0.09. A double peak in the histogram of the activation energy was found, and the activation of the crystallization energy was calculated as E_A_ = 220 kJ/mol. The basics thermal parameters were determined as T_g_ = (521, 524, and 532 °C), T_x_ = (682, 697, and 714 °C), and ∆T = (161, 173, and 182 °C). The glass transformation temperatures changed from 521 to 532 °C by increasing the heating rate. The glass-ceramic structure with the Na_6_Ca_3_(Si_6_O_18_) (combeite) phase was obtained by the controlled heat treatment of the host glass. The formed nanocrystals were calculated from Scherrer’s formula and their size was approximately 29 nm. The biological properties of the glass-ceramics evaluated in SBF for 7 days showed that there was no negative influence on the formation of the hydroxy-carbonate apatite (HCA) layer. The structure of the material was observed and analyzed by doping the 45S5 Bioglass with Eu^3+^ ions and by measuring the changes of the luminescence profile. A decreasing fluorescence intensity ratio indicated surrounding symmetry around lanthanide ions. The doped glass fibers were produced by two methods, which allowed a comparison of the structure. Significant differences were noted in this range, which confirmed the crystallization of fibers during the standard method of fiber drawing. 

## Figures and Tables

**Table 1:** Crystallization peak temperatures of 45S5 Bioglass^®^ for different heating rates.

Rate β (°C/min)	Tg (°C)	Tx (°C)	∆T (°C)
5	521	682	161
10	524	697	173
15	532	714	182

**Table 2:** Avrami parameters calculated at each heating rate.

β °C/min	5	10	15	Avg.	SD
n	1.53	1.42	1.34	1.43	0.09

**Table 3:** Center assignment [35,36,37,38,39,40,41,42,43,44].

Center (cm^−1^)	Assignment Vibration
1150	asymmetric stretching vibrations of the Si=O and P=O
1100–1000	asymmetric stretching vibrations of the asymmetric Si-O-Si
1000–1050	asymmetric stretching vibrations of the asymmetric Si-O(P)
1040–840	asymmetric stretching vibrations of the asymmetric Si-O- and P-O-
800–730	symmetric stretching vibrations of the Si-O(Si) and Si-O(P) bridging bonds
720–590	silico‒oxygen rings of different numbers of ring members
550–400	bending vibrations of the O-P-O and O-Si-O bridges

**Table 4:** Parameters of the component bands of the MIR spectrum of the 45S5_0h sample [35,36,37,38,39,40,41,42,43,44].

Center (cm^−1^)	Area	Height	FWHM	Assignment Vibration
450	13.88	0.19	64.31	bending vibrations of O-Si-O and O-P-O
515	43.36	0.43	89.47	
596	1.68	0.05	27.72	6-membered silico‒oxygen ring vibrations
678	9.26	0.11	72.61	3- and 4-membered silico‒oxygen rings vibrations
735	6.16	0.10	53.29	symmetric stretching vibrations of the Si-O(Si) and Si-O(P)
839	27.78	0.33	75.05	asymmetric stretching vibrations of the Si-O^-^ and P-O^-^
915	65.73	0.59	98.84	
1004	30.12	0.29	92.50	asymmetric stretching Si-O(P) vibrations
1059	61.78	0.40	135.41	asymmetric stretching Si-O(Si) vibrations
1139	53.45	0.23	200.13	asymmetric stretching Si=O and P=O vibrations

**Table 5:** Parameters of the component bands of the MIR spectrum of the 45S5_4h sample [35,36,37,38,39,40,41,42,43,44].

Center (cm^−1^)	Area	Height	FWHM	Assignment Vibration
453	52.29	0.77	60.34	bending vibrations of O-Si-O and O-P-O
515	96.79	1.14	76.07	
606	6.45	0.15	37.60	6-membered silico‒oxygen ring vibrations
686	15.97	0.20	69.58	3- and 4-membered silico‒oxygen rings vibrations
734	6.43	0.15	37.48	symmetric stretching vibrations of the Si-O(Si) and Si-O(P)
839	51.16	0.74	61.42	asymmetric stretching vibrations of the Si-O^-^ and P-O^-^
915	207.15	1.76	105.34	
1011	104.16	1.08	86.50	asymmetric stretching Si-O(P) vibrations
1072	150.53	1.19	113.34	asymmetric stretching Si-O(Si) vibrations
1148	83.37	0.53	138.89	asymmetric stretching Si=O and P=O vibrations
